# 427. Underlying Medical Conditions and Vaccination Status among U.S. Children Aged ≤17 Years Hospitalized for COVID-19 in COVID-19-Associated Hospitalization Surveillance Network (COVID-NET), January–December 2023

**DOI:** 10.1093/ofid/ofae631.141

**Published:** 2025-01-29

**Authors:** Rebecca J Free, Kadam Patel, Darpun Sachdev, Nisha B Alden, James Meek, Patricia A Ryan, Anna Falkowski, Paige D’Heilly, Chad Smelser, Kerianne Engesser, Sophrena Bushey, Nancy E Moran, Melissa Sutton, H Keipp Talbot, Ashley Swain, Angela P Campbell, Fiona Havers, Kyle P Openo

**Affiliations:** Centers for Disease Control and Prevention, Atlanta, GA; Centers for Disease Control and Prevention, Atlanta, GA; California Department of Public Health, Richmond, California; Colorado Department of Public Health and Environment, Denver, Colorado; Connecticut Emerging Infections Program, Yale School of Public Health, New Haven, Connecticut; Maryland Department of Health, Baltimore, Maryland; Michigan Department of Health and Human Services, Livonia, Michigan; Minnesota Department of Health, St. Paul, Minnesota; New Mexico Department of Health, Santa Fe, New Mexico; New York State Department of Health, Albany, New York; University of Rochester School of Medicine and Dentistry, Rochester, New York; Ohio Dept of Health, Columbus, Ohio; Oregon Health Authority, Portland, Oregon; Vanderbilt University, Nashville, TN; Salt Lake County Health Department, Salt Lake City, Utah; Centers for Disease Control and Prevention, Atlanta, GA; Centers for Disease Control and Prevention, Atlanta, GA; Georgia Emerging Infections Program and Atlanta VA Medical Center, Decatur, GA

## Abstract

**Background:**

SARS-CoV-2 infection can result in serious illness in children. We used COVID-19-Associated Hospitalization Surveillance Network (COVID-NET) data to assess characteristics associated with pediatric COVID-19 hospitalizations, including underlying medical conditions and vaccination status.

**Figure:**
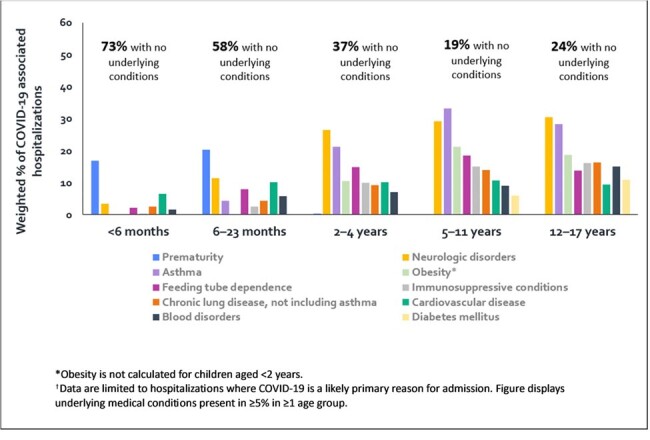

Percent of COVID-19-Associated Hospitalizations† with Specific Underlying Medical Conditions among U.S. Children Aged ≤17 Years by Age Group — COVID-NET, January–December 2023

**Methods:**

U.S. children aged ≤ 17 years with COVID-19 as the likely primary reason for hospitalization during January–December 2023 were identified in the population-based COVID-NET active surveillance system, which includes patients residing in a catchment area of > 300 acute care hospitals in 13 states with a positive SARS-CoV-2 test result during or ≤ 14 days before admission. Medical records of a random sample of children stratified by age-group and site were abstracted; reported percentages were weighted to account for probability of selection. We assessed patient age, underlying medical conditions, and receipt of vaccine dose within 12 months before illness.

**Figure:**
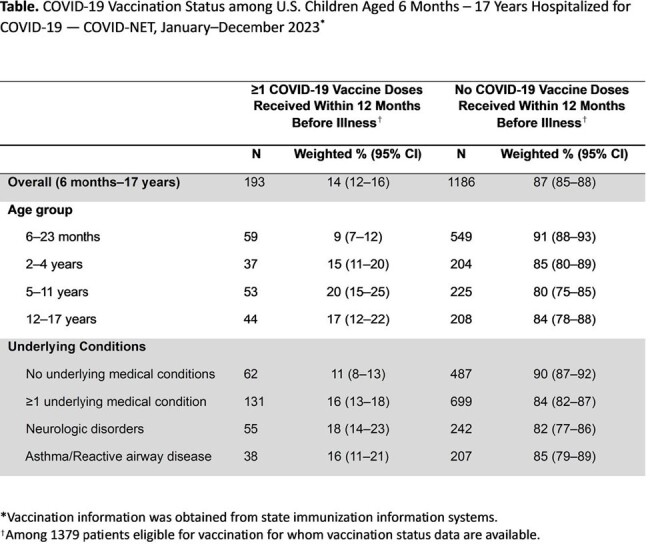

**Results:**

Of 2016 children hospitalized for COVID-19, 1232 (61%) were aged < 2 years (611 aged < 6 months; 621 aged 6–23 months). Overall, 51% had no underlying condition, including 73% of infants aged < 6 months, 58% of children aged 6–23 months, and 24% of children aged 12–17 years (Figure). Among children aged < 2 years, prematurity was the most common underlying condition (17–20%). Neurologic disorders (27–31%) and asthma (21–33%) were most prevalent among children aged ≥ 2 years. Among 1379 children aged ≥ 6 months eligible for vaccination with vaccine data available, only 14% had received ≥ 1 vaccine dose within 12 months before illness, with the lowest frequency (9%) among children aged 6–23 months (Table). Among age-eligible children with neurologic disorders and asthma, only 18% and 16% were vaccinated, respectively.

**Conclusion:**

Most children aged < 2 years hospitalized for COVID-19 during 2023 had no underlying conditions; underlying conditions were more prevalent among older children. More than 85% of age-eligible children had not received a vaccine dose within 12 months, including among children with underlying conditions. Increasing pediatric COVID-19 vaccination, especially among children with underlying medical conditions, could help reduce COVID-19 hospitalizations.

**Disclosures:**

**Anna Falkowski, Master of Science**, Council of State and Territorial Epidemiologists (CSTE): Grant/Research Support

